# Ultrasound-guided diagnostic breast biopsy methodology: retrospective comparison of the 8-gauge vacuum-assisted biopsy approach versus the spring-loaded 14-gauge core biopsy approach

**DOI:** 10.1186/1477-7819-9-87

**Published:** 2011-08-11

**Authors:** Stephen P Povoski, Rafael E Jimenez, Wenle P Wang

**Affiliations:** 1 Division of Surgical Oncology, Department of Surgery, Arthur G. James Cancer Hospital and Richard J. Solove Research Institute and Comprehensive Cancer Center, The Ohio State University, Columbus, Ohio, 43210, USA; 2 Department of Pathology, The Ohio State University, Columbus, Ohio, 43210, USA; 3 Division of Anatomic Pathology, Department of Laboratory Medicine and Pathology, Mayo Clinic, Rochester, Minnesota, 55905, USA; 4 Department of Pathology, VA Medical Center at Baltimore, Baltimore, Maryland, 21201, USA

## Abstract

**Background:**

Ultrasound-guided diagnostic breast biopsy technology represents the current standard of care for the evaluation of indeterminate and suspicious lesions seen on diagnostic breast ultrasound. Yet, there remains much debate as to which particular method of ultrasound-guided diagnostic breast biopsy provides the most accurate and optimal diagnostic information. The aim of the current study was to compare and contrast the 8-gauge vacuum-assisted biopsy approach and the spring-loaded 14-gauge core biopsy approach.

**Methods:**

A retrospective analysis was done of all ultrasound-guided diagnostic breast biopsy procedures performed by either the 8-gauge vacuum-assisted biopsy approach or the spring-loaded 14-gauge core biopsy approach by a single surgeon from July 2001 through June 2009.

**Results:**

Among 1443 ultrasound-guided diagnostic breast biopsy procedures performed, 724 (50.2%) were by the 8-gauge vacuum-assisted biopsy technique and 719 (49.8%) were by the spring-loaded 14-gauge core biopsy technique. The total number of false negative cases (i.e., benign findings instead of invasive breast carcinoma) was significantly greater (P = 0.008) in the spring-loaded 14-gauge core biopsy group (8/681, 1.2%) as compared to in the 8-gauge vacuum-assisted biopsy group (0/652, 0%), with an overall false negative rate of 2.1% (8/386) for the spring-loaded 14-gauge core biopsy group as compared to 0% (0/148) for the 8-gauge vacuum-assisted biopsy group. Significantly more (P < 0.001) patients in the spring-loaded 14-gauge core biopsy group (81/719, 11.3%) than in the 8-gauge vacuum-assisted biopsy group (18/724, 2.5%) were recommended for further diagnostic surgical removal of additional tissue from the same anatomical site of the affected breast in an immediate fashion for indeterminate/inconclusive findings seen on the original ultrasound-guided diagnostic breast biopsy procedure. Significantly more (P < 0.001) patients in the spring-loaded 14-gauge core biopsy group (54/719, 7.5%) than in the 8-gauge vacuum-assisted biopsy group (9/724, 1.2%) personally requested further diagnostic surgical removal of additional tissue from the same anatomical site of the affected breast in an immediate fashion for a benign finding seen on the original ultrasound-guided diagnostic breast biopsy procedure.

**Conclusions:**

In appropriately selected cases, the 8-gauge vacuum-assisted biopsy approach appears to be advantageous to the spring-loaded 14-gauge core biopsy approach for providing the most accurate and optimal diagnostic information.

## Background

It is well established among breast health care professionals that ultrasound-guided diagnostic breast biopsy technology represents the current recommended standard of care for accomplishment of the most minimally invasive evaluation of indeterminate and suspicious lesions seen on diagnostic breast ultrasound [1-3]. Nevertheless, there remains much debate as to which particular method of ultrasound-guided diagnostic breast biopsy provides the most accurate and optimal diagnostic information [4-10]. In this regard, there seems to be an increasing trend towards the use of larger-gauged vacuum-assisted biopsy technology for ultrasound-guided diagnostic breast biopsies [4-77], particularly by the 8-gauge vacuum-assisted biopsy approach [7,8,19,20,22,27,28,31,35,36,40,41,44-47,49-54,56-58,60-62,65-68,70,74,75]. The purpose of the current report is to retrospectively compare and contrast the results of two ultrasound-guided diagnostic breast biopsy methodologies, the 8-gauge vacuum-assisted biopsy approach and the spring-loaded 14-gauge core biopsy approach, amongst a large series of ultrasound-guided diagnostic breast biopsy procedures performed by a single surgeon.

## Methods

This retrospective study was approved by the Clinical Scientific Review Committee and by the Cancer Institutional Review Board of The Arthur G. James Cancer Hospital and Richard J. Solove Research Institute and Comprehensive Cancer Center of The Ohio State University Medical Center.

All patients who underwent an ultrasound-guided diagnostic breast biopsy by a single surgeon (SPP) using an 8-gauge vacuum-assisted biopsy device or a spring-loaded 14-gauge core biopsy device from the time period of July 2001 through June 2009 were identified. All of the ultrasound-guided diagnostic breast biopsy procedures were performed at The James Comprehensive Breast Center of The Arthur G. James Cancer Hospital and Richard J. Solove Research Institute and Comprehensive Cancer Center of The Ohio State University Medical Center. These ultrasound-guided diagnostic breast biopsy procedures were all performed using freehand real-time ultrasound guidance with high-resolution linear array transducers, as previously described [8,40]. The 8-gauge vacuum-assisted biopsies were performed using the 8-gauge Mammotome^® ^breast biopsy system (Devicor Medical Products, Inc., Cincinnati, Ohio). The spring-loaded 14-gauge core biopsies were performed using either the Achieve^® ^spring-loaded 14-gauge core biopsy device (Cardinal Health, Inc., McGraw Park, Illinois) or the Bard^® ^MaxCore™ spring-loaded 14-gauge core biopsy device (C.R. Bard, Inc., Covington, Georgia).

All of the breast lesions undergoing ultrasound-guided diagnostic breast biopsy were sonographically visible and were classified according to the American College of Radiology (ACR) Breast Imaging Reporting and Data System (BI-RADS) as either BI-RADS category 3, 4, or 5. All BI-RADS category 4 and 5 ultrasound breast lesions were strongly recommended for ultrasound-guided diagnostic breast biopsy. For those ultrasound breast lesions classified as BI-RADS category 4 and 5, pre-biopsy mammography was obtained when it was determined appropriate, as based upon patient age and clinical indications. However, for those ultrasound breast lesions classified as BI-RADS category 4 and 5, further pre-biopsy diagnostic breast imaging with magnetic resonance imaging was not considered. As a general rule, the vast majority of BI-RADS category 3 ultrasound breast lesions seen at The James Comprehensive Breast Center were recommended for serial short-term patient follow-up alone, consisting of repeat clinical breast examination and repeat diagnostic breast imaging at an interval of time of 3 to 6 months after the designation of an ultrasound breast lesion as BI-RADS category 3. However, ultrasound-guided diagnostic breast biopsy was performed on BI-RADS category 3 ultrasound breast lesions when the patient expressed concern and the desire for having a diagnostic breast biopsy rather than having serial short-term patient follow-up alone.

For the 8-gauge vacuum-assisted biopsy procedures, local anesthetic, consisting of 1% lidocaine plain (used for the skin and superficial tissues, and ranging from 5 to 15 mL) and 1% lidocaine containing 1:100,000 mixture of epinephrine (used for the deeper breast tissues surround the ultrasound lesion, and ranging from 15 to 30 mL), was utilized. For the spring-loaded 14-gauge core biopsy procedures, local anesthetic, consisting of only 1% lidocaine plain (ranging from 15 to 30 mL), was utilized. After local anesthetic was administered, a #11 blade was used to make an approximately 5 mm skin incision entrance site for the 8-gauge vacuum-assisted biopsy procedures and an approximately 2 mm skin incision entrance site for the spring-loaded 14-gauge core biopsy procedures. Further details with regard to the specific techniques used during the 8-gauge vacuum-assisted biopsy procedures have been previously reported [8,40]. After the completion of core acquisition and after the removal of ultrasound-guided diagnostic biopsy device from the breast, a 14-gauge Cormark™ rigid microclip device (Devicor Medical Products, Inc., Cincinnati, Ohio) was inserted under ultrasound guidance through the same breast parenchymal track for placement of a microclip into the area of the ultrasound-guided diagnostic biopsy. Placement of a microclip was done selectively for ultrasound-guided diagnostic breast biopsy procedures performed from 2001 to 2004, but was generally done more universally thereafter.

Manual compression to the breast was generally performed for approximately ten minutes after completion of the ultrasound-guided diagnostic breast biopsy procedure to assure adequate hemostasis to the biopsy site. The skin incision entrance site was then generally closed with either adhesive skin closure strips or absorbable suture. In selected cases, a circumferential compressive ace wrap was applied to the chest of patients for a post-procedural duration of approximately 24 hours.

All submitted ultrasound-guided diagnostic breast biopsy core specimens were processed in the Department of Surgical Pathology for permanent histopathologic evaluation with routine Hematoxylin and Eosin (H&E) staining. All information with regards to the histopathologic diagnosis was obtained from the official pathology report issued by the Department of Surgical Pathology.

The histopathologic findings from each of the original ultrasound-guided diagnostic breast biopsy procedures were generally first discussed by telephone with the patients at the soonest availability of those pathology results. All patients with abnormal histopathologic findings on pathologic evaluation that clinically warranted surgical intervention were appropriately counseled and recommended for such management. The demonstration of a biopsy-proven neoplasm on the original ultrasound-guided diagnostic breast biopsy was generally recommended for immediate therapeutic surgical excision. The demonstration of an indeterminate or inconclusive finding on the original ultrasound-guided diagnostic breast biopsy was generally recommended for immediate diagnostic surgical excision. Indeterminate or inconclusive finding included high risk breast lesions (i.e., atypical ductal hyperplasia, atypical lobular hyperplasia, or lobular carcinoma in situ) seen on the original ultrasound-guided diagnostic biopsy, as well as clinical or radiographic suspicion in any given case which was out of proportion of the of benign findings seen on the original ultrasound-guided diagnostic breast biopsy (i.e., the results of the original ultrasound-guide diagnostic biopsy do not seem to explain the original lesion seen on breast imaging). All patients with benign findings on histopathologic evaluation were asked to return for interval breast-related patient follow-up, generally consisting of clinical breast examination and breast imaging (consisting of ultrasound and/or mammography) at an initial recommended follow-up time interval of approximately 6 months after the time of the original ultrasound-guided diagnostic breast biopsy procedure. There was variability in the timing of interval breast-related patient follow-up for many patients with benign pathology secondary to patient availability issues and patient compliance issues. Some patients with benign pathology remained completely noncompliant, and, resultantly, had no interval breast-related patient follow-up, even after multiple attempts to arrange such follow-up. There was also variability in the performance of interval follow-up breast imaging, primarily based upon patients' personal preferences for undergoing such interval follow-up breast imaging. Some patients with benign findings on the original ultrasound-guided diagnostic breast biopsy procedure themselves requested an immediate surgical excision procedure.

Finally, if interval follow-up breast imaging showed abnormal findings for which an interval, repeat diagnostic breast biopsy procedure was recommended or if patients themselves requested an interval, repeat diagnostic breast biopsy procedure despite stable interval follow-up breast imaging, then an interval, repeat diagnostic breast biopsy procedure was performed in a delayed fashion.

The data collection of all variables was accomplished by way of retrospective review of The Ohio State University Medical Center's electronic medical records system. Multiple variables, including patient demographics, lesion variables, procedural variables, histopathology variables, and interval breast-related patient follow-up variables, were evaluated. Interval breast-related patient follow-up was last updated as of March 2011.

The histopathology results from the biopsy core specimens harvested at the time of each original ultrasound-guided diagnostic breast biopsy procedure were assessed in comparison to the final histopathologic diagnosis rendered in each case, and including: (1) those instances in which further therapeutic or diagnostic surgical removal of additional tissue from the same anatomical site of the affected breast was done in an immediate fashion after the original ultrasound-guided diagnostic breast biopsy procedure; (2) those instances in which patient-requested surgical removal of additional tissue from the same anatomical site of the affected breast was done in an immediate fashion after having benign findings on the original ultrasound-guided diagnostic breast biopsy procedure; (3) those instances in which a subsequent, interval, repeat diagnostic breast biopsy procedure was later done in a delayed fashion to the same anatomical site of the affected breast as results of an abnormality noted on interval follow-up breast imaging at the time of interval breast-related patient follow-up; and (4) those instances in which a patient-requested subsequent, interval, repeat diagnostic breast biopsy procedure was later done in a delayed fashion at the time of interval breast-related patient follow-up to the same anatomical site of the affected breast after previously having benign findings on the original ultrasound-guided diagnostic breast biopsy procedure and after having stable interval follow-up breast imaging at the time of interval breast-related patient follow-up. This assessment process was done in order to determine the misestimation of any given breast finding, the overall number of false negative findings, and the overall false negative rate. The determination of the misestimation of any given breast finding, as it pertained to benign breast findings, high risk breast lesions, ductal carcinoma in situ (DCIS), DCIS with microinvasion, and invasive carcinoma, was made for the original ultrasound-guided diagnostic breast biopsy procedure findings as a direct comparison to the final histopathologic diagnosis for all cases in which subsequent therapeutic or diagnostic removal of additional tissue from the same anatomical site of the affected breast was performed in an immediate fashion. The determination of the overall number of false negative results was made from the entire population of each group for all patients who returned for some form of interval breast-related patient follow-up by comparing the original ultrasound-guided diagnostic breast biopsy procedure results to that of the final determination of the status of the affected breast, as based upon those instances in which subsequent removal of additional tissue from the same anatomical site of the affected breast was performed in both an immediate fashion and a delayed fashion, as well as based upon final determination of the status of the affected breast of all other cases in each group not undergoing subsequent removal of additional tissue from the affected breast but who returned for some form of interval breast-related patient follow-up. A false negative finding was specifically defined as any instance in which an ultrasound lesion, initially shown to be benign at the time of the original ultrasound-guided diagnostic breast biopsy procedure, was subsequently shown to be a carcinoma (i.e., invasive carcinoma or DCIS) on any further subsequent removal (in an immediate fashion or in a delayed fashion) of additional tissue from the same anatomical site of the affected breast. Additionally, the false negative rate for the identification of a carcinoma (i.e., invasive carcinoma or DCIS) was calculated from the equation of the number of the false negative results divided by the sum of the number of the true positive results and the number of the false negative results.

The software program IBM SPSS^® ^19 for Windows^® ^(SPSS, Inc., Chicago, Illinois) was used for all statistical analyses. For univariate comparisons of categorical variables, either Pearson chi-square test or Fisher exact test was utilized. Continuous variables were expressed as median (range) or mean (± standard deviation) or both, when appropriate. For univariate comparisons of continuous variables, one-way analysis of variance (ANOVA) was utilized. All univariate P-values that were determined to be 0.05 or less were considered to be significant. All reported P-values were two-sided.

## Results

Patient demographics and characteristics of the original breast lesions are shown in Table 1 for all patients undergoing an ultrasound-guided diagnostic breast biopsy procedure. Of the 1443 ultrasound-guided diagnostic breast biopsy procedures performed, 724 (50.2%) were performed by the 8-gauge vacuum-assisted biopsy technique and 719 (49.8%) were performed by the spring-loaded 14-gauge core biopsy technique. Patients undergoing an 8-gauge vacuum-assisted biopsy had a predilection toward having smaller-sized (median 1.10 cm, range 0.28-5.53), nonpalpable lesions that were more frequently classified as either BI-RADS category 4 (607/724, 83.8%) or BI-RADS category 3 (78/724, 10.8%). Whereas, patients undergoing a spring-loaded 14-gauge core biopsy had a predilection toward having larger-sized (median 2.00 cm, range 0.42-9.08), palpable lesions that were more frequently classified as either BI-RADS category 4 (523/719, 72.4%) or BI-RADS category 5 (177/719, 24.6%).

**Table 1:** Patient demographics and characteristics of the original breast lesions in all cases of ultrasound-guided diagnostic breast biopsy (8-gauge vacuum-assisted biopsy or spring-loaded 14-gauge core biopsy).

	8-gauge	14-gauge	All cases	P-value
Total number of cases	724	719	1443	-----------
				
Age (median, years)	50 (18-87)	49 (18-96)	49 (18-96)	0.498
				
Gender				0.823
Female	713 (98.5%)	710 (98.7%)	1423 (98.6%)	
Male	11 (1.5%)	9 (1.3%)	20 (1.4%)	
				
Breast				0.599
Right	347 (47.9%)	355 (49.4%)	702 (48.6%)	
Left	377 (52.1%)	364 (50.6%)	741 (51.4%)	
				
Palpable tumor				<0.001
Yes	288 (39.8%)	561 (78.0%)	849 (58.8%)	
No	436 (60.2%)	158 (22.0%)	594 (41.2%)	
				
Lesion location				0.201
UOQ	364 (50.3%)	402 (55.9%)	766 (53.1%)	
UIQ	155 (21.4%)	124 (17.2%)	279 (19.3%)	
LOQ	115 (15.9%)	105 (14.6%)	220 (15.2%)	
LIQ	58 (8.0%)	54 (7.5%)	112 (7.8%)	
Subareolar	32 (4.4%)	34 (4.7%)	66 (4.6%)	
				
BI-RADS classification on ultrasound				<0.001
Category 3	78 (10.8%)	19 (2.6%)	97 (6.7%)	
Category 4	607 (83.8%)	523 (72.7%)	1130 (78.3%)	
Category 5	39 (5.4%)	177 (24.6%)	216 (15.0%)	
				
Lesion size on ultrasound (median, cm)	1.10 (0.28-5.53)	2.00 (0.42-9.08)	1.50 (0.28-9.08)	<0.001

UOQ, upper outer quadrant; LOQ, lower outer quadrant; UIQ, upper inner quadrant; LIQ, lower inner quadrant; BI-RADS, breast imaging reporting and data system

Procedural variables are shown in Table 2 for all patients undergoing an ultrasound-guided diagnostic breast biopsy procedure. Although, at first glance, the median number of core removed at the time of the ultrasound-guided diagnostic breast biopsy appeared to be the same for the 8-gauge vacuum-assisted biopsy group (6 cores, range 1 to 38) as compared to the spring-loaded 14-gauge core biopsy group (6 cores, range 2 to 15), the mean number of core removed was determined to actually be significantly greater (P < 0.001) for the 8-gauge vacuum-assisted biopsy group (7.6 ± 5.1) as compared to the spring-loaded 14-gauge core biopsy group (6.0 ± 2.1). However, as is shown in Table 2, this finding of the statistical analysis for the number of cores removed at the time of the ultrasound-guided diagnostic breast biopsy was purely a reflection of the impact of the number of cores removed at the time of those 8-gauge vacuum-assisted diagnostic biopsy procedures that were also done with the intention to attempt 8-gauge vacuum-assisted excision of any given benign breast lesion (median = 8, range 1 to 38; mean = 9.3 ± 5.9, N = 354). This was further exemplified by the fact that when one looked solely at those individuals with a final diagnosis of breast cancer, the median and mean number of cores removed at the time of the ultrasound-guided diagnostic breast biopsy appeared to be similar to or to even have a near-opposite trend (i.e., a borderline, but non-significant P-value of 0.087) for the 8-gauge vacuum-assisted biopsy group (median = 4, range 2 to 22; mean = 5.5 ± 3.6, N = 148) as compared to the spring-loaded 14-gauge core biopsy group (median = 6, range 2 to 15; mean = 6.0 ±2.2, N = 386).

**Table 2:** Procedural variables for all cases of ultrasound-guided diagnostic breast biopsy (8-gauge vacuum-assisted biopsy or spring-loaded 14-gauge core biopsy).

	8-gauge	14-gauge	All cases	P-value
Total number of cases	724	719	1443	-----------
				
Number of cores (all procedures)				<0.001
Median (range)	6.0 (1-38)	6.0 (2-15)	6.0 (1-38)	
Mean (±SD)	7.6 (±5.1)	6.0 (±2.1)	6.8 (±4.0)	
				
Number of cores (with final pathology as carcinoma)				0.087
Median (range)	4.0 (2-22)	6.0 (2-15)	5.0 (2-22)	
Mean (±SD)	5.5 (±3.6)	6.0 (±2.2)	5.9 (±2.6)	
				
Number of cores (with final pathology as benign)^#^				<0.001
Median (range)	7.0 (1-38)	6.0 (2-14)	6.0 (1-38)	
Mean (±SD)	8.2 (±5.4)	6.0 (±2..0)	7.4 (±4.6)	
				
Placement of marking microclip*				<0.001
Yes	714 (98.6%)	428 (59.5%)	1142 (79.1%)	
No	10 (1.4%)	291 (40.5%)	301 (20.9%)	

^# ^For those 8-gauge vacuum-assisted diagnostic biopsies that were done with the intention to attempt 8-gauge vacuum-assisted excision of any given benign breast lesions (n = 354), the median number of cores was 8 (range, 1 to 38) and the mean number of cores was 9.3 (± 5.9).

* Microclip marking was done selectively for ultrasound-guided diagnostic breast biopsy procedures done during the time period from 2001 to 2004, but was generally done more universally in all cases thereafter.

The diagnosis from the histopathology evaluation of the breast biopsy core specimens harvested at the time of each original ultrasound-guided diagnostic breast biopsy procedure for all cases are shown in Table 3.

**Table 3:** Histopathology from the breast biopsy core specimens harvested at the time of the original ultrasound-guided diagnostic breast biopsy procedure.

	8-gauge	14-gauge	All cases
Total number of cases	724	719	1443
Carcinomas^#^	148 (20.4%)	377 (52.4%)	525 (36.4%)
High risk breast lesions^#^	15 (2.1%)	6 (0.8%)	21 (1.5%)
Fibroadenomas	239 (33.0%)	147 (20.4%)	386 (26.7%)
Benign breast changes/conditions^†^	261 (36.0%)	145 (20.2%)	406 (28.1%)
Intraductal papillomas	42 (5.8%)	6 (0.8%)	48 (3.3%)
Indeterminate fibroepithelial breast lesions	0 (0%)	8 (1.1%)	8 (0.6%)
Benign phyllodes tumors	1 (0.1%)	1 (0.1%)	2 (0.1%)
Malignant phyllodes tumors	0 (0%)	0 (0%)	0 (0%)
Lymphomas/leukemias	4 (0.6%)	7 (1.0%)	11 (0.8%)
Benign lymphoid tissue	13 (1.8%)	21 (2.9%)	34 (2.4%)
Desmoids/fibromatosis	1 (0.1%)	1 (0.1%)	2 (0.1%)

# carcinomas included invasive carcinoma and ductal carcinoma in situ (DCIS).

* high risk breast lesions included atypical ductal hyperplasia, atypical lobular hyperplasia, and lobular carcinoma in situ.

† benign breast changes/conditions included all of the following histopathologic terminologies issued in official pathology report from Department of Surgical Pathology: fibrocystic breast changes, ductal epithelial hyperplasia, sclerosing adenosis, stromal fibrosis, cyst-formation, ductal ectasia, fibrous mastopathy, lymphocytic mastopathy, diabetic mastopathy, columnar cell changes, fat necrosis, hemorrhage, scar-formation, gynecomastia, adenosis tumor, lactating adenoma, hamartoma, lipoma, myofibroblastoma, amyloidosis, benign granular cell tumor, epidermal inclusion cyst, or benign breast tissue with no pathologic changes.

Post-procedural complications are shown in Table 4 for all patients undergoing an ultrasound-guided diagnostic breast biopsy procedure. Both the overall number of post-procedural complications and the individual type of post-procedural complications were not significantly different (P = 0.810 and P = 0.922, respectively) for the 8-gauge vacuum-assisted biopsy group versus the spring-loaded 14-gauge core biopsy group. For neither the 8-gauge vacuum-assisted biopsy group nor the spring-loaded 14-gauge core biopsy group was there the need of subsequent intraoperative surgical management of any resultant post-procedural complication. Interestingly, for the entire group of 1443 patients undergoing an ultrasound-guided diagnostic breast biopsy procedure, patients with a diagnosis of carcinoma on the original ultrasound-guided diagnostic breast biopsy procedure were more likely (P < 0.001) to have a post-procedural bleeding complication (93/525, 17.7%) than were patients without a diagnosis of carcinoma on the original ultrasound-guided diagnostic breast biopsy procedure (82/918, 8.9%). Also, interestingly, for the entire group of 525 with a diagnosis of carcinoma on the original ultrasound-guided diagnostic breast biopsy procedure, there was no significant difference (P = 0.284) in the overall frequency of occurrence of a post-procedural bleeding complication for the 8-gauge vacuum-assisted biopsy group (22/148, 14.9%) as compared to for the spring-loaded 14-gauge core biopsy group (71/377, 18.8%). Nevertheless, if one looked at the occurrence of a post-procedural bleeding complication separately for the 8-gauge vacuum-assisted biopsy group and for the spring-loaded 14-gauge core biopsy group as a function of having a diagnosis of carcinoma made at the time of the original ultrasound-guided diagnostic breast biopsy procedure, one noted that patients undergoing a spring-loaded 14-gauge core biopsy procedure were more likely (P < 0.001) to have a post-procedural bleeding complication with a diagnosis of carcinoma on the original ultrasound-guided diagnostic breast biopsy procedure (71/377, 18.8%) than without a diagnosis of carcinoma on the original ultrasound-guided diagnostic breast biopsy procedure (18/342, 5.3%), while patients undergoing an 8-gauge vacuum-assisted biopsy procedure were not more likely (P = 0.208) to have a post-procedural bleeding complication with a diagnosis of carcinoma on the original ultrasound-guided diagnostic breast biopsy procedure (22/148, 14.9%) than without a diagnosis of carcinoma on the original ultrasound-guided diagnostic breast biopsy procedure (64/576, 11.1%).

**Table 4:** Post-procedural complications for all cases of ultrasound-guided diagnostic breast biopsy (8-gauge vacuum-assisted biopsy or spring-loaded 14-gauge core biopsy).

	8-gauge	14-gauge	All cases	P-value
Post-procedural complication				0.810
Yes	87 (12.0%)	90 (12.5%)	177 (12.3%)	
No	637 (88.0%)	629 (87.5%)	1266 (87.7%)	
				
Type of post-procedural complication				0.922
Mild hematoma/skin ecchymosis	70 (9.7%)	69 (9.6%)	139 (9.6%)	
Moderate hematoma/skin ecchymosis	16 (2.2%)	20 (2.8%)	36 (2.5%)	
Severe hematoma/skin ecchymosis	0 (0%)	0 (0%)	0 (0%)	
Infectious complication	1 (0.1%)	1 (0.1%)	2 (0.1%)	

The further therapeutic or diagnostic surgical removal of additional tissue from the same anatomical site of the affected breast and patient-requested surgical removal of additional tissue from the same anatomical site of the affected breast done in an immediate fashion after the original ultrasound-guided diagnostic breast biopsy procedure is shown in Table 5. Overall, for all the ultrasound-guided diagnostic breast biopsy procedures performed, further diagnostic or therapeutic removal of additional tissue from the same anatomical site of the affected breast was recommended more frequently (P < 0.001) in the group undergoing a spring-loaded 14-gauge core biopsy procedure (515/719, 71.6%) as compared to the group undergoing an 8-gauge vacuum-assisted biopsy procedure (180/724, 24.9%). Most notably, this was a direct consequence of the fact that 379/719 (52.7%) of the spring-loaded 14-gauge core biopsy procedures yielded a biopsy-proven neoplasm that were recommended for immediate therapeutic surgical excision while only 153/724 (21.1%) of the 8-gauge vacuum-assisted biopsy procedures yielded a biopsy-proven neoplasm that was recommended for immediate therapeutic surgical excision (P < 0.001). Nevertheless, significantly more (p < 0.001) of the spring-loaded 14-gauge core biopsy procedures (81/719, 11.3%) showed an indeterminate or inconclusive finding that was recommended for immediate diagnostic surgical excision to the affected breast than did the 8-gauge vacuum-assisted biopsy procedures (18/724, 2.5%). Similarly, in significantly more cases (P < 0.001), patients undergoing a spring-loaded 14-gauge core biopsy procedure that showed a biopsy-proven benign breast finding (54/719, 7.5%) requested an immediate diagnostic surgical excision of that biopsy-proven benign breast finding than did patients undergoing an 8-gauge vacuum-assisted biopsy that showed a biopsy-proven benign finding (9/724, 1.2%). This was possibly a consequence of the fact that median lesion size of biopsy-proven benign breast findings in patients requesting immediate diagnostic surgical excision of such biopsy-proven benign breast findings was significantly larger (P < 0.001) in the spring-loaded 14-gauge core biopsy group (2.60 cm, range 0.57-7.02) than in the 8-gauge vacuum-assisted biopsy group (0.50 cm, range 0.32-1.20).

**Table 5:** Further therapeutic or diagnostic surgical removal of additional tissue from the same anatomical site of the affected breast and patient-requested surgical removal of additional tissue from the same anatomical site of the affected breast, done in an immediate fashion, after the original ultrasound-guided diagnostic breast biopsy procedure.

	8-gauge	14-gauge	All cases	P-value
All cases in which there was a recommendation for further therapeutic or diagnostic surgical removal of additional tissue from the affected breast, or the patient personally requested surgical removal of additional tissue from the affected breast in an immediate fashion	180 (24.9%)	515 (71.6%)	695 (48.2%)	<0.001
All cases in which the previous recommendation for further therapeutic or diagnostic surgical removal of additional tissue from the affected breast was not subsequently undertaken	9 (5.0%)	44 (8.5%)	53 (7.6%)	0.123
All cases in which there was a recommendation for further therapeutic surgical removal of additional tissue from the affected breast in an immediate fashion for a biopsy-proven neoplasm	153 (21.1%)	379 (52.7%)	532 (36.9%)	<0.001
All cases in which the previous recommendation for further therapeutic surgical removal of additional tissue from the affected breast for a biopsy-proven neoplasm was not subsequently undertaken	4 (2.6%)	41 (10.8%)	45 (8.5%)	0.002
Reason why previous recommendation for further therapeutic surgical removal of additional tissue from the affected breast for a biopsy-proven neoplasm was not subsequently undertaken				---------
Co-existing distant metastatic disease	2 (50.0%)	23 (56.1%)	25 (55.6%)	---------
Co-morbid conditions	1 (25.0%)	13 (31.7%)	14 (31.1%)	---------
Patient elected to pursue treatment elsewhere	1 (25.0%)	5 (12.2%)	6 (13.3%)	---------
All cases in which there was a recommendation for further diagnostic surgical removal of additional tissue from the affected breast in an immediate fashion for an indeterminate/inconclusive finding on the original ultrasound-guided diagnostic breast biopsy	18 (2.5%)	81 (11.3%)	99 (6.9%)	<0.001
All cases in which the previous recommendation for further diagnostic surgical removal of additional tissue from the affected breast in an immediate fashion for an indeterminate/inconclusive finding on the original ultrasound-guided diagnostic breast was not subsequently undertaken	5 (27.8%)	3 (3.7%)	8 (8.1%)	0.005
Reason why previous recommendation for further diagnostic surgical removal of additional tissue from the affected breast for an indeterminate/inconclusive finding on the original ultrasound-guided diagnostic breast was not subsequently undertaken				---------
Patient preferred observation alone	3 (60.0%)	0 (0%)	3 (37.5%)	---------
Patient elected to pursue treatment elsewhere	2 (40.0%)	3 (100%)	5 (62.5%)	---------
All cases in which the patient personally requested further diagnostic surgical removal of additional tissue from the affected breast in an immediate fashion after having a benign finding on the original ultrasound-guided diagnostic breast biopsy	9 (1.2%)	54 (7.5%)	63 (4.4%)	<0.001

An assessment of the accuracy of the original ultrasound-guided diagnostic breast biopsy by 8-gauge vacuum-assisted biopsy technique versus by spring-loaded 14-gauge core biopsy technique for all cases in which a subsequent surgical excision of additional tissue from the same anatomical site of the affected breast was performed in an immediate fashion is shown in Table 6. Overall, the histopathologic finding on the initial ultrasound-guided diagnostic breast biopsy matched exactly to the final histopathologic diagnosis on a subsequent immediate surgical excision of tissue from the same anatomical site of the affected breast more frequently (P < 0.001) for the 8-gauge vacuum-assisted biopsy group (168/171, 98.2%) than for the spring-loaded 14-gauge core biopsy group (410/471, 87.0%). Significantly more (P < 0.001) of the spring-loaded 14-gauge core biopsy results (37/471, 7.9%) showed a mismatch in the type of benign diagnosis as compared to the 8-gauge vacuum-assisted biopsy results (0/171, 0%). Although not statistically significant (P = 0.199), more misestimations of benign findings instead of invasive carcinoma were observed for the spring-loaded 14-gauge core biopsy group (7/471, 1.5%) than for the 8-gauge vacuum-assisted biopsy group (0/171, 0%) after a subsequent surgical excision of additional tissue from the same anatomical site of the affected breast was performed in an immediate fashion.

**Table 6:** Assessment of accuracy of the initial ultrasound-guided diagnostic breast biopsy by 8-gauge vacuum-assisted biopsy technique versus spring-loaded 14-gauge core biopsy technique for all cases in which a subsequent surgical excision of tissue from the same anatomical site of the affected breast was performed in an immediate fashion.

	8-gauge	14-gauge	All cases	P-value
Cases in which a subsequent surgical excision of tissue from the affected breast was performed in an immediate fashion	171 (23.6%)	471 (65.5%)	642 (44.5%)	<0.001
Histopathologic findings matched exactly for both the initial ultrasound-guided biopsy and the subsequent immediate surgical excision	168 (98.2%)	410 (87.0%)	578 (90.0%)	<0.001
Mismatch observed in the type of benign diagnosis	0 (0%)	37 (7.9%)	37 (5.8%)	<0.001
Misestimation of benign findings instead of invasive carcinoma	0 (0%)	7 (1.5%)	7 (1.1%)	0.199
Misestimation of benign findings instead of DCIS with microinvasive	0 (0%)	0 (0%)	0 (0%)	---------
Misestimation of benign findings instead of DCIS	0 (0%)	0 (0%)	0 (0%)	---------
Misestimation of high-risk breast lesions instead of invasive carcinoma	0 (0%)	0 (0%)	0 (0%)	---------
Misestimation of high-risk breast lesions instead of DCIS with microinvasive	0 (0%)	0 (0%)	0 (0%)	---------
Misestimation of high-risk breast lesions instead of DCIS	0 (0%)	1 (0.2%)	1 (0.2%)	1.0
Misestimation of DCIS instead of invasive carcinoma	1 (0.6%)	6 (1.3%)	7 (1.1%)	0.682
Misestimation of DCIS instead of DCIS with microinvasion	2 (1.2%)	0 (0%)	2 (0.3%)	0.071

* high risk breast lesions included atypical ductal hyperplasia, atypical lobular hyperplasia, and lobular carcinoma in situ. DCIS: ductal carcinoma in situ

Interval breast-related patient follow-up variables are shown in Table 7. Over 90% of patients in both the 8-gauge vacuum-assisted biopsy group (N = 652) and the spring-loaded 14-gauge core biopsy group (N = 681) had some form of interval breast-related patient follow-up. For all patients in each group who returned for some form of interval breast-related patient follow-up, the median duration of the last interval breast-related patient follow-up was greater than 26 months. For those patient in each group who had benign biopsy results on the original ultrasound-guided diagnostic breast biopsy and who were not recommended for or requested having a subsequent immediate diagnostic or therapeutic surgical excision of additional tissue and who returned for some form of interval breast-related patient follow-up, the median duration of the last interval breast-related patient follow-up was greater than 24 months.

**Table 7:** Interval breast-related patient follow-up variables.

	8-gauge	14-gauge	All cases	P-value
Did the patient return for any interval breast-related patient follow-up?				0.001
Yes	652 (90.1%)	681 (94.7%)	1333 (92.4%)	
No	72 (9.9%)	38 (5.3%)	110 (7.6%)	
				
Median duration to last interval breast-related patient follow-up visit for all patients in each group (months, range)	26.3 (0.4-101.5)	32.1 (0.3-113.2)	28.5 (0.3-113.2)	<0.001
				
Median duration to last interval breast-related patient follow-up visit for those patients in each group who had a benign biopsy result and who were not recommended for or requested having a subsequent immediate surgical excision (months, range)	24.6 (1.9-101.5)	24.4 (1.2-96.9)	24.5 (1.2-101.5)	0.034

Subsequent, interval, repeat diagnostic breast biopsy procedures done in a delayed fashion to the same anatomical site of the affected breast after having a benign finding on the original ultrasound-guided diagnostic breast biopsy procedure are shown in Table 8. There was no difference (P = 0.211) in the frequency at which an interval, repeat diagnostic breast biopsy procedure (i.e., diagnostic, imaged-guided, minimally-invasive breast biopsy or diagnostic surgical excision) was done in a delayed fashion to the affected breast after the original ultrasound-guided diagnostic breast biopsy procedure showed benign findings for the group undergoing a spring-loaded 14-gauge core biopsy procedure (15/719, 2.1%) as compared to the group undergoing an 8-gauge vacuum-assisted biopsy procedure (9/724, 1.2%). The reasons for these interval, repeat diagnostic breast biopsy procedures and the type of these interval, repeat diagnostic breast biopsy procedures are shown in Table 8. In one single case, a benign breast finding from the initial ultrasound-guided diagnostic breast biopsy for the spring-loaded 14-gauge core biopsy group was determined to actually represent an invasive carcinoma at the time of the interval, repeat diagnostic breast biopsy procedure done in a delayed fashion.

**Table 8:** Subsequent, interval, repeat diagnostic breast biopsy procedures that were later done in a delayed fashion from the same anatomical site of the affected breast after having a benign finding on the original ultrasound-guided diagnostic breast biopsy procedure.

	8-gauge	14-gauge	All cases	P-value
All cases in which the patient underwent an interval, repeat diagnostic breast biopsy procedure done at a delayed time after having a benign finding on the original ultrasound-guided diagnostic breast biopsy	9 (1.2%)	15 (2.1%)	24 (1.7%)	0.211
Median time to interval, repeat diagnostic breast biopsy procedure (months, range)	12.4 (4.7-45.4)	9.8 (2.8-34.1)	12.0 (2.8-45.4)	0.373
Reason for interval, repeat diagnostic breast biopsy procedure				---------
Residual BIRADS 4 ultrasound lesion	6 (66.7%)	7 (46.7%)	12 (50.0%)	---------
Residual BIRADS 4 MRI lesion	1 (11.1%)	0 (0%)	1 (4.2%)	---------
Developed new BIRADS 4 mammographic lesion	2 (22.2%)	0 (0%)	2 (8.3%)	---------
Patient's request	0 (0%)	8 (53.3%)	8 (33.3%)	---------
Type of interval, repeat diagnostic breast biopsy procedure				---------
Surgical excision	3 (33.3%)	7 (46.7%)	10 (41.7%)	---------
Ultrasound-guided 8-gauge vacuum-assisted biopsy	5 (55.5%)	7 (46.7%)	12 (50.0%)	---------
Ultrasound-guided 14-gauge core biopsy	0 (0%)	1 (6.7%)	1 (4.2%)	---------
MRI guided 10-gauge biopsy	1 (11.1%)	0 (0%)	1 (4.2%)	---------
Frequency in which a benign breast finding from the original ultrasound-guided diagnostic breast biopsy was determined to represent an invasive carcinoma at the time of the interval, repeat diagnostic breast biopsy procedure done at a delayed time	0/9 (0%)	1/15 (6.7%)	1/24 (4.2%)	1.000

The final histopathologic diagnosis for all cases, which included any changes made in the final histopathologic diagnosis as a result of all instances in which subsequent diagnostic removal of additional tissue from the same anatomical site of the affected breast was performed in an immediate fashion or in a delayed fashion, is shown in Table 9.

**Table 9:** Final histopathologic diagnosis, including all instances in which subsequent diagnostic removal of tissue from the same anatomical site of the affected breast was performed in an immediate fashion or in a delayed fashion.

	8-gauge	14-gauge	All cases
Total number of cases	724	719	1443
Carcinomas^#^	148 (20.4%)	386 (53.7%)	534 (37.0%)
High risk breast lesions^#^	15 (2.1%)	5 (0.7%)	20 (1.4%)
Fibroadenomas	238 (32.9%)	151 (21.0%)	389 (27.0%)
Benign breast changes/conditions^†^	261 (36.0%)	138 (19.2%)	399 (27.7%)
Intraductal papillomas	42 (5.8%)	6 (0.8%)	48 (3.3%)
Indeterminate fibroepithelial breast lesions	0 (0%)	0 (0%)	0 (0%)
Benign phyllodes tumors	2 (0.3%)	4 (0.6%)	6 (0.4%)
Malignant phyllodes tumors	0 (0%)	1 (0.1%)	1 (0.1%)
Lymphomas/leukemias	4 (0.6%)	7 (1.0%)	11 (0.8%)
Benign lymphoid tissue	13 (1.8%)	20 (2.8%)	33 (2.3%)
Desmoids/fibromatosis	1 (0.1%)	1 (0.1%)	2 (0.1%)

# carcinomas included invasive carcinoma and ductal carcinoma in situ.

* high risk breast lesions included atypical ductal hyperplasia, atypical lobular hyperplasia, and lobular carcinoma in situ.

† benign breast changes/conditions included all of the following histopathologic terminologies issued in official pathology report from Department of Surgical Pathology: fibrocystic breast changes, ductal epithelial hyperplasia, sclerosing adenosis, stromal fibrosis, cyst-formation, ductal ectasia, fibrous mastopathy, lymphocytic mastopathy, diabetic mastopathy, columnar cell changes, fat necrosis, hemorrhage, scar-formation, gynecomastia, adenosis tumor, lactating adenoma, hamartoma, lipoma, myofibroblastoma, amyloidosis, benign granular cell tumor, epidermal inclusion cyst, or benign breast tissue with no pathologic changes.

Overall, for those patients who returned for some form of interval breast-related patient follow-up (N = 1333), the total number of false negative results, as defined as an initial ultrasound-guided diagnostic breast biopsy showing benign findings but a subsequent removal of additional tissue from the same anatomical site of the affected breast (done in either an immediate fashion or a delayed fashion) showing breast carcinoma, was significantly greater (P = 0.008) in the spring-loaded 14-gauge core biopsy group (8/681, 1.2%) as compared to in the 8-gauge vacuum-assisted biopsy group (0/652, 0%). In all eight cases, this represented a missed invasive breast carcinoma. This translates into an overall false negative rate for the identification of an invasive breast carcinoma of 2.1% (8/386) for the spring-loaded 14-gauge core biopsy group as compared to 0% (0/148) for the 8-gauge vacuum-assisted biopsy group. There was no apparent relationship noted between the size of the ultrasound lesion originally biopsied by the ultrasound-guided spring-loaded 14-gauge core biopsy approach to that of the overall false negative rate, since no difference (P = 0.786) was demonstrated in the median lesion size for those eight cases of a false negative result (2.36 cm, range 0.91-3.00) from the spring-loaded 14-gauge core biopsy group as compared to the entire spring-loaded 14-gauge core biopsy group (2.00 cm, range 0.42-9.08). However, as expected, there was a marginal relationship (P = 0.059) between the BI-RADS classification and the total number of false negative results in the spring-loaded 14-gauge core biopsy procedure group for those individuals who returned for some form of interval breast-related patient follow-up (N = 681), with 0 false negative results in 19 patients (0%) who had a BI-RADS category 3 lesion on their initial ultrasound, versus 3 false negative results in 485 patients (0.6%) who had a BI-RADS category 4 lesion on their initial ultrasound, versus 5 false negative results in 177 patients (2.8%) who had a BI-RADS category 5 lesion on their initial ultrasound.

For the patients evaluated in this study during the time period from July 2001 through June 2009, two patients in the spring-loaded 14-gauge core biopsy procedure group and three patients in the 8-gauge vacuum-assisted biopsy procedure group subsequently developed a breast cancer event in a different anatomical site of the ipsilateral breast that was geographically separate from the location of the original ultrasound-guided diagnostic breast biopsy procedure. These events occurred at 27 months and 29 months after the original ultrasound-guided diagnostic breast biopsy for the two spring-loaded 14-gauge core biopsy patients and occurred at 9 months, 48 months, and 56 months after the original ultrasound-guided diagnostic breast biopsy for the three 8-gauge vacuum-assisted biopsy patients.

## Discussion

When carefully scrutinizing the data from our currently reported series, several important findings become apparent with regards to the methodology of ultrasound-guided diagnostic breast biopsy. First, and foremost, when specifically looking at all of the patients who underwent some form of interval breast-related patient follow-up (N = 1333), the total number of false negative cases (i.e., benign findings instead of invasive breast carcinoma) was found to be significantly greater (P = 0.008) in the spring-loaded 14-gauge core biopsy group (8/681, 1.2%) as compared to in the 8-gauge vacuum-assisted biopsy group (0/652, 0%). This translates into an overall false negative rate for the identification of an invasive breast carcinoma of 2.1% (8/386) for the spring-loaded 14-gauge core biopsy group as compared to 0% (0/148) for the 8-gauge vacuum-assisted biopsy group. Second, significantly more (P < 0.001) patients in the spring-loaded 14-gauge core biopsy group (81/719, 11.3%) than in the 8-gauge vacuum-assisted biopsy group (18/724, 2.5%) were recommended for further diagnostic surgical removal of additional tissue from the same anatomical site of the affected breast in an immediate fashion for indeterminate/inconclusive findings seen on the original ultrasound-guided diagnostic breast biopsy procedure. Third, significantly more (P < 0.001) patients in the spring-loaded 14-gauge core biopsy group (54/719, 7.5%) than in the 8-gauge vacuum-assisted biopsy group (9/724, 1.2%) personally requested further diagnostic surgical removal of additional tissue from the same anatomical site of the affected breast in an immediate fashion for a benign finding seen on the original ultrasound-guided diagnostic breast biopsy procedure. Collectively, these findings support the use of 8-gauge vacuum-assisted biopsy technology over that of spring-loaded 14-gauge core biopsy technology for ultrasound-guided diagnostic breast biopsy procedure in appropriately selected cases.

As is shown in Table 10, there is an abundance of studies in the literature reporting on the false negative rate for the spring-loaded 14-gauge core biopsy approach [4,6,78-95]. However, there is a relative paucity of information available in the literature that specifically addresses the accurate determination of the false negative rate for the 8-gauge vacuum-assisted biopsy approach. In our currently reported series, the overall false negative rate for finding an invasive breast carcinoma by the spring-loaded 14-gauge core biopsy approach was 2.1% (8/386). This is highly consistent with the cumulative results of the false negative rate, as shown in Table 10, for the spring-loaded 14-gauge core biopsy approach that have been previously reported by many other authors [4,6,78-95]. This determination and comparison is very helpful for validating the skill-set level of the surgeon in the currently reported series who performed all of the ultrasound-guided diagnostic breast biopsies, both by the spring-loaded 14-gauge core biopsy technique and by the 8-gauge vacuum-assisted biopsy technique. In this specific regard, the overall false negative rate for the identification of an invasive breast carcinoma in the currently reported series by the 8-gauge vacuum-assisted biopsy approach was 0%. This represent a series of 724 patients undergoing an 8-gauge vacuum-assisted biopsy procedures, in which 652 of those patients underwent some form of interval breast-related patient follow-up, and which constituted a total of 148 cases in which a breast carcinoma was diagnosed by the 8-gauge vacuum-assisted biopsy approach. To date, this represents the largest reported series of breast carcinomas diagnosed by the 8-gauge vacuum-assisted biopsy technique and the most comprehensive evaluation of the efficacy of the 8-gauge vacuum-assisted biopsy approach.

**Table 10:** Studies specifically reporting on the false negative rate of identifying breast malignancies based upon the ultrasound-guided 14-gauge core diagnostic breast biopsy approach.

Citation [reference]	False negative rate
Parker 1993 [78]	0% (0/34)
Nguyen 1996 [79]	2.1% (4/187)
Liberman 2000 [80]	3.7% (9/241)
Schoonjans 2001 [81]	1.7% (4/243)
Buchberger 2002 [82]	1.3% (3/234)
Memarsadeghi 2003 [83]	3.1% (5/161)
Philpotts 2003 [4]	2.8% (1/36)
Shah 2003 [84]	3.6% (3/84)
Delle Chiaie 2004 [85]	3.1% (4/128)
Fajardo 2004 [86]	2.6% (2/77)
Pijnappel 2004 [87]	11.8% (8/68)
Cho 2005 [6]	3.1% (4/128)
Crystal 2005 [88]	3.1% (10/323)
Dillon 2005 [89]	1.7% (13/769)
Sauer 2005 [90]	2.3% (14/618)
Vega Bolivar 2005 [91]	3.3% (4/122)
Wu 2006 [92]	2.4% (5/209)
Fan 2008 [93]	1.1% (18/1584)
Schueller 2008 [94]	1.6% (11/709)
Youk 2009 [95]	2.5% (50/1982)

Cumulative results	2.2% (172/7937)

Povoski 2011	2.1% (8/386)

In the currently reported series, the statistical analysis demonstrates that there was some degree of inherent selection bias created by the surgeon regarding the decision-making process as to whether the 8-gauge vacuum-assisted biopsy approach or the spring-loaded 14-gauge core biopsy approach was utilized for performing any given ultrasound-guided diagnostic breast biopsy procedure. Specifically, there was a predilection toward utilizing the 8-gauge vacuum-assisted biopsy approach for smaller-sized ultrasound lesions (i.e., those less than 1.0 cm to 1.5 cm in greatest dimension), nonpalpable lesions, and/or lesions that were classified as either BI-RADS category 4 or 3; whereas, there was a predilection toward utilizing the spring-loaded 14-gauge core biopsy approach for larger-sized ultrasound lesions (i.e., those greater than 2.0 cm to 2.5 cm in greatest dimension), palpable lesions, and/or lesions that were classified as either BI-RADS category 4 or 5. Hence, this demonstrates an inherent but understandable selection bias for utilizing the spring-loaded 14-gauge core biopsy technique for ultrasound lesions which appear to represent more obvious breast cancers, based upon their larger size and their more suspicious appearance on diagnostic breast imaging, and is supported by the findings in the current reported series in which 52.4% (377/719) of the ultrasound lesions approached by the spring-loaded 14-gauge core biopsy technique at the time of the original ultrasound-guided diagnostic breast biopsy procedure were breast carcinomas while only 20.4% (148/724) of the ultrasound lesions approached by the 8-gauge vacuum-assisted biopsy technique were breast carcinomas.

Despite this inherent but understandable selection bias for utilizing the spring-loaded 14-gauge core biopsy technique for ultrasound lesions which appear to represent more obvious breast cancers, there are several less obvious but still very key points that are worth mentioning with regards to appropriate patient selection for whether the 8-gauge vacuum-assisted biopsy approach or the spring-loaded 14-gauge core biopsy approach is utilized for performing any given ultrasound-guided diagnostic breast biopsy procedure. While these key points may embody the opinion of the authors of the currently reported series, they are potentially useful to all breast health care professionals that utilize ultrasound-guided diagnostic breast biopsy technology.

The first less obvious but still key point relates to the issue of the adequacy of tissue sampling for small, subcentimeter, but highly suspicious (i.e., BI-RADS category 4 or 5) ultrasound lesions [8]. There is always an inherent degree of uncertainty that exists within one's mind when using the spring-loaded 14-gauge core biopsy technique secondary to concerns about positional overshooting or undershooting that may occur with the tissue acquisition chamber when firing the spring-loaded 14-gauge core biopsy device when attempting to target any such small, subcentimeter ultrasound lesion. In contrast, approaching such small, subcentimeter ultrasound lesions by the 8-gauge vacuum-assisted biopsy technique allows for more representative and even potentially complete tissue sampling of any given small, subcentimeter region of interest in a more precise and directed fashion. This line of reasoning was utilized in the currently reported series in which 39.2% (58/148) of breast carcinomas diagnosed by the 8-gauge vacuum-assisted biopsy technique were less than 1 cm in size, while only 4.9% (19/386) of breast carcinomas diagnosed by the spring-loaded 14-gauge core biopsy technique were less than 1 cm in size (P < 0.001). Such an approach may be highly advantageous for helping to potentially minimize the risks for misestimation of any given breast finding and for reducing the risks of false negative results for finding a breast carcinoma at the time of ultrasound-guided diagnostic breast biopsy.

The second less obvious but still key point relates to the issue of tissue sampling of larger-sized but vaguely characterized areas within the breast that may be of clinical concern and/or radiographic concern. Several examples of this scenario easily come to mind and include: (1) attempting to differentiate diabetic mastopathy from that of a breast carcinoma; (2) attempting to differentiate scar tissue and post-surgical changes within the breast from that of a breast carcinoma; and (3) attempting to differentiate severe fibrocystic breast changes from that specifically of invasive lobular carcinoma. In these particular situations, a spring-loaded 14-gauge core biopsy approach to ultrasound-guided diagnostic breast biopsy may prove highly difficult due to the inability for the tissue acquisition chamber of the spring-loaded 14-gauge core biopsy device to correctly and completely fire through such breast tissue of increased breast tissue density that is frequently encountered in these particular situations. Likewise, in these particular situations, a spring-loaded 14-gauge core biopsy approach may create concerns about the certainty of the degree of representative tissue sampling of a larger-sized but vaguely characterized area of interest within the breast. On the other hand, in these particular situations, an 8-gauge vacuum-assisted biopsy approach to ultrasound-guided diagnostic breast biopsy would allow for single-pass, central placement of the device within such a larger-sized but vaguely characterized area of interest within the breast, despite the finding of generalized increased breast tissue density, with subsequent ease of tissue acquisition of multiple 8-gauge cores in up to a complete 360 degree rotational array. Resultantly, this would allow for the achievement of large-volume and highly representative tissue sampling of the entire larger-sized but vaguely characterized area of interest within the breast, thus maximizing the accuracy of tissue diagnosis and reducing the potential risks of false negative results for correctly identifying a breast carcinoma.

## Conclusions

The 8-gauge vacuum-assisted biopsy approach to ultrasound-guided diagnostic breast biopsy appears to be advantageous to that of the spring-loaded 14-gauge core biopsy approach for providing the most accurate and optimal diagnostic information. In appropriately selected patients, the 8-gauge vacuum-assisted biopsy approach can: (1) minimize the overall false negative rate for diagnosing invasive breast carcinoma; (2) reduce the subsequent need for further diagnostic removal of additional tissue from the affected breast in an immediate fashion for indeterminate/inconclusive findings; and (3) reduce patient-requested further diagnostic removal of additional tissue from the affected breast in an immediate fashion despite benign finding the original ultrasound-guided diagnostic breast biopsy procedure. The importance of appropriate patient selection for either of these ultrasound-guided diagnostic breast biopsy approaches can not be underestimated and must be well understood by those breast health care professionals utilizing ultrasound-guided diagnostic breast biopsy technology.

## Competing interests

The article-processing charge for this manuscript was paid for by Devicor Medical Products, Inc. (Cincinnati, Ohio). The authors declare that they have no other relevant competing interests to disclose.

## Authors' contributions

**SPP **was the surgeon who performed all the ultrasound-guided diagnostic biopsy procedures. He designed the current study, collected the data, and performed all data analyses. He organized, wrote, and edited all aspects of this manuscript. **REJ **and **WPW **were the pathologists who were involved in reading the histopathology for many of the cases contained within this series and were involved in writing and editing this manuscript. All of the authors have read and approved the final version of this manuscript.
